# MYC–MAX heterodimerization is essential for the induction of major zygotic genome activation and subsequent preimplantation development

**DOI:** 10.1038/s41598-023-43127-5

**Published:** 2023-09-25

**Authors:** Takuto Yamamoto, Haoxue Wang, Hana Sato, Shinnosuke Honda, Shuntaro Ikeda, Naojiro Minami

**Affiliations:** Laboratory of Reproductive Biology, Graduate School of Agriculture, Kyoto University, Kyoto, 606-8502 Japan

**Keywords:** Embryogenesis, Embryology

## Abstract

In mouse preimplantation development, zygotic genome activation (ZGA), which synthesizes new transcripts in the embryo, begins in the S phase at the one-cell stage, with major ZGA occurring especially at the late two-cell stage. *Myc* is a transcription factor expressed in parallel with ZGA, but its direct association with major ZGA has not been clarified. In this study, we found that developmental arrest occurs at the two-cell stage when mouse embryos were treated with antisense oligonucleotides targeting *Myc* or MYC-specific inhibitors from the one-cell stage. To identify when MYC inhibition affects development, we applied time-limited inhibitor treatment and found that inhibition of MYC at the one-cell, four-cell, and morula stages had no effect on preimplantation development, whereas inhibitor treatment at the two-cell stage arrested development at the two-cell stage. Furthermore, transcriptome analysis revealed that when MYC function was inhibited, genes expressed in the major ZGA phase were suppressed. These results suggest that MYC is essential for the induction of major ZGA and subsequent preimplantation development. Revealing the function of MYC in preimplantation development is expected to contribute to advances in assisted reproductive technology.

## Introduction

The *Myc* family of transcription factors consists of *Myc*, *Mycn*, and *Mycl*. In most cases, these components form a heterodimer with Myc-associated factor X (MAX) via the leucine zipper domain and bind to target E-boxes for transcription elongation^1,2^. In mammals, a newly fertilized embryo uses mRNAs and proteins stored in the cytoplasm of the oocyte to sustain subsequent development^3–5^. In the mouse embryo, these maternal mRNAs and proteins are degraded as development progresses, and two rounds of zygotic genome activation (ZGA), called minor and major ZGA, initiate the synthesis of new transcripts in the embryo. The former occurs during 8–10 h post-insemination (hpi) at the one-cell stage, while the latter occurs late in the two-cell stage^6–9^. *Myc* is known to be an α-amanitin sensitive gene in one- to two-cell stage embryos, indicating that *Myc* is one of the ZGA genes^10^. Immunofluorescence staining analysis has demonstrated that MYC protein is detected in nuclear speckles, which control pre-mRNA splicing immediately after transcription. MYC in nuclear speckles is localized in the nucleus in growing oocytes and fully grown oocytes as well as in fertilized eggs (embryos) up to the morula stage, but signal intensity is slightly weakened at the morula stage and no signal is observed at the blastocyst stage^11^. Only a few studies have attempted to determine the function of *Myc* in preimplantation development by inhibition, but these studies have produced inconsistent results due to different timing of inhibition and different molecular targets.

It has been reported that when *Myc* mRNA is degraded by antisense oligonucleotides (ASOs) from the two-cell stage, developmental arrest occurs at the eight-cell/morula stage^12^. Homozygous mutations in *Myc* are lethal between embryonic days 9.5 and 10.5^13^. It has also been reported that inhibition of MYC–MAX dimerization by inhibitors of MYC from 2 h after in vitro fertilization results in the suppression of minor ZGA and developmental arrest mainly at the one-cell stage^14^. However, the risk of nonspecific effects should always be considered when using inhibitors. These reports indicate that development can proceed to embryonic day 9.5 with maternally expressed *Myc* alone, and that inhibition of both maternal and embryonic *Myc* results in developmental arrest during preimplantation development. However, because the previous study inhibited MYC from the two-cell stage, when ZGA has already begun, and used only inhibitors, the possibility that the phenotype was an artifact could not be ruled out, and thus the exact role of MYC in preimplantation development could not be determined.

In this study, we treated one-cell stage embryos with *Myc*-targeting ASOs or MYC-specific inhibitors and investigated their subsequent development. Furthermore, after determining when inhibitors affected embryonic development, we also examined the effect of MYC inhibition on gene expression.

## Results

### Knockdown of Myc mRNA in one-cell embryos results in embryonic lethality at the two-cell stage

**Figure 1:**
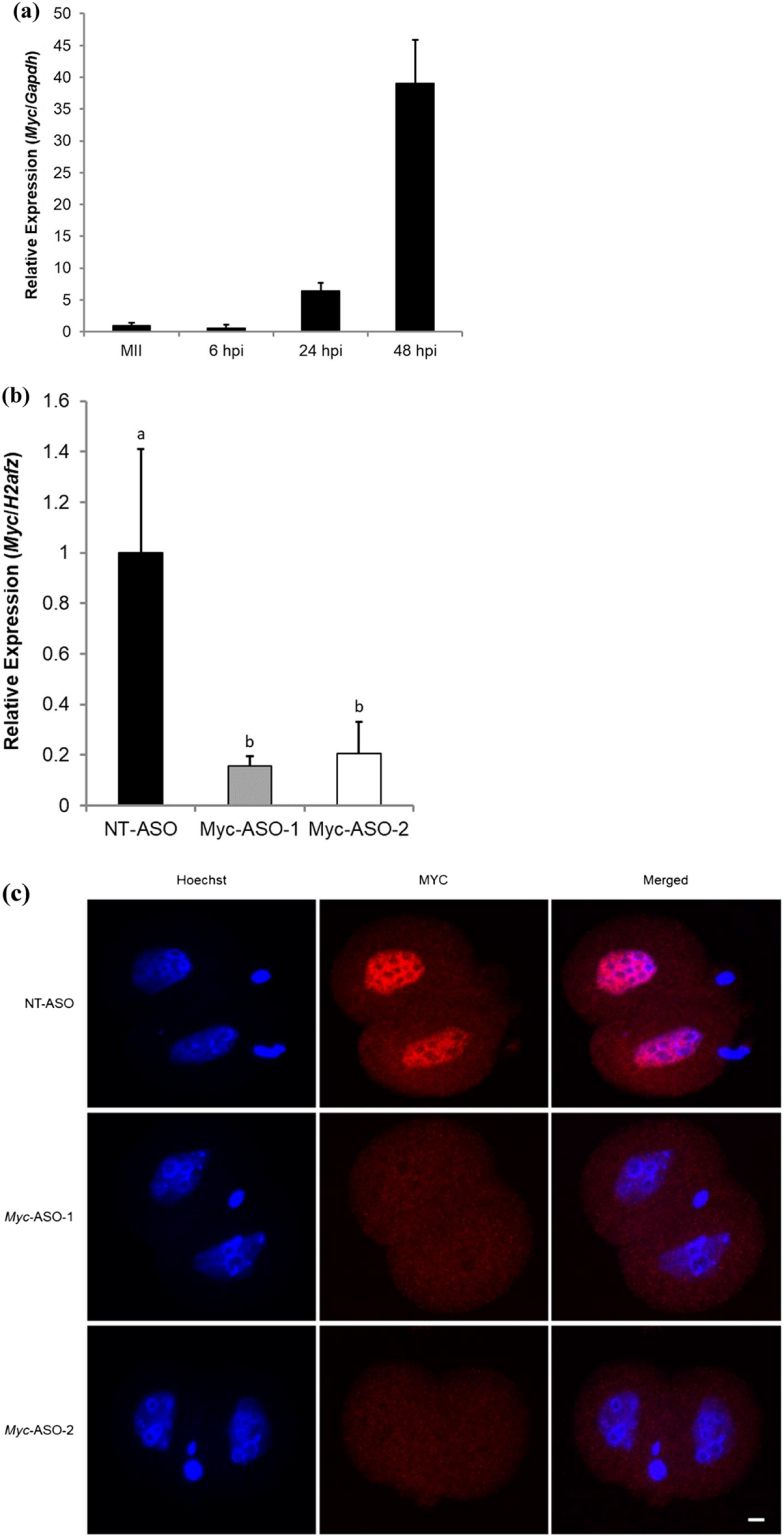

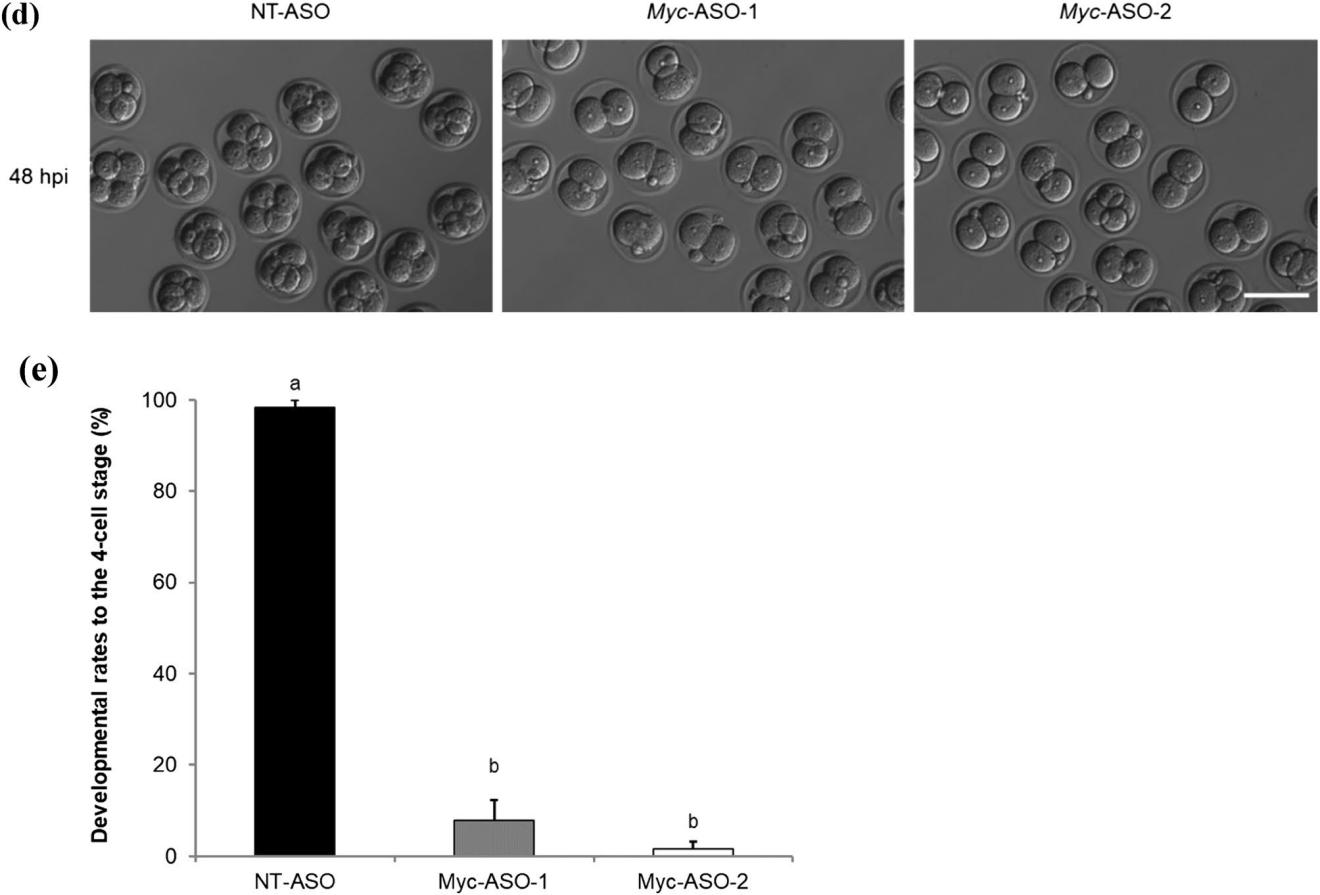
Effects of inhibiting *Myc* mRNA expression on preimplantation development. (**a**) RT-qPCR analysis of *Myc* mRNA expression in metaphase II (MII) oocytes and preimplantation embryos. Gene expression levels were normalized to *Gapdh* as an internal control. Data are expressed as the mean ± standard error of the mean (SEM) (*n* = 3). Thirty oocytes or embryos were analyzed in each treatment. (**b**) RT-qPCR analysis of *Myc* mRNA expression in *Myc*-knockdown embryos and control embryos at 24 hpi. Gene expression levels were normalized to *H2afz* as an internal control. Data are expressed as the mean ± SEM (*n* = 3). Fifteen embryos were analyzed in each treatment. Statistical analysis was performed using one-way analysis of variance (ANOVA) followed by the Tukey–Kramer test. *P*-values < 0.05 were considered statistically significant and are represented by different letters (a and b). (**c**) Localization of MYC in embryos injected with either *Myc*-ASO-1 (*n* = 16), *Myc*-ASO-2 (*n* = 15), or NT-ASO (*n* = 12) at the one-cell stage. MYC was localized in the nuclei of embryos injected with NT-ASO. However, MYC nuclear localization disappeared in embryos injected with *Myc*-ASOs. Embryos were photographed at 24 hpi. Scale bar, 20 μm. (**d**) Representative images of embryos injected with either *Myc*-ASO-1, *Myc*-ASO-2, or NT-ASO at the one-cell stage. Embryos were photographed at 48 hpi. Scale bar, 100 μm. (**e**) Developmental rates of embryos injected with either *Myc*-ASO-1 (*n* = 63), *Myc*-ASO-2 (*n* = 62), or NT-ASO (*n* = 58) at the one-cell stage. Data are expressed as the mean ± SEM (*n* = 3). Statistical analysis was performed using the chi-squared test with Holm’s adjustment. *P*-values < 0.05 were considered statistically significant and are represented by different letters (a and b).

The expression pattern of *Myc* mRNA in mouse metaphase II oocytes and preimplantation embryos was examined. Reverse transcription-quantitative PCR (RT-qPCR) analysis of *Myc* mRNA showed that its expression increased from 6 to 48 hpi (Fig. 1a), consistent with a previous report analyzing published RNA-sequencing (seq) data^15^. To investigate the role of *Myc* in early embryonic development, *Myc* was knocked down in mouse preimplantation embryos. *Myc*-targeting ASOs (*Myc*-ASO-1 and *Myc*-ASO-2) were microinjected into embryos at 3 hpi and cultured until 96 hpi. RT-qPCR confirmed a significant decrease in *Myc* mRNA expression at 24 hpi (Fig. 1b), and immunofluorescence staining showed that MYC was not incorporated into the nuclei of *Myc*-knocked down embryos at 24 hpi (Fig. 1c). Morphological observation showed that the rate of development of *Myc*-ASO-microinjected embryos to the four-cell stage was markedly reduced compared with control embryos (98.3% for non-targeted control [NT]-ASO, 7.9% for *Myc*-ASO-1, and 1.6% for *Myc*-ASO-2 at 48 hpi) (Fig. 1d and e).

### MYC–MAX heterodimerization is essential for development at the two-cell stage

**Figure 2:**
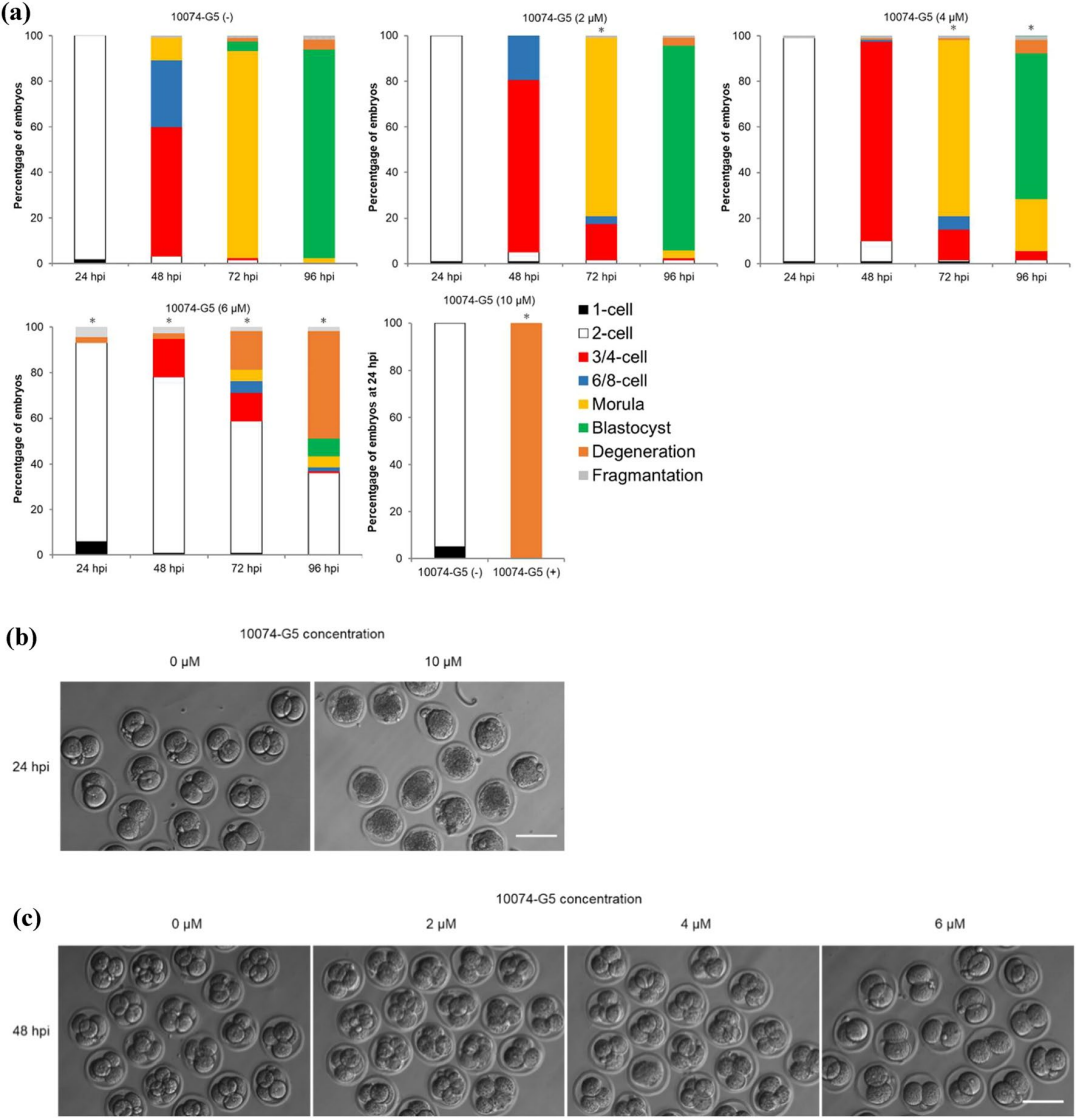

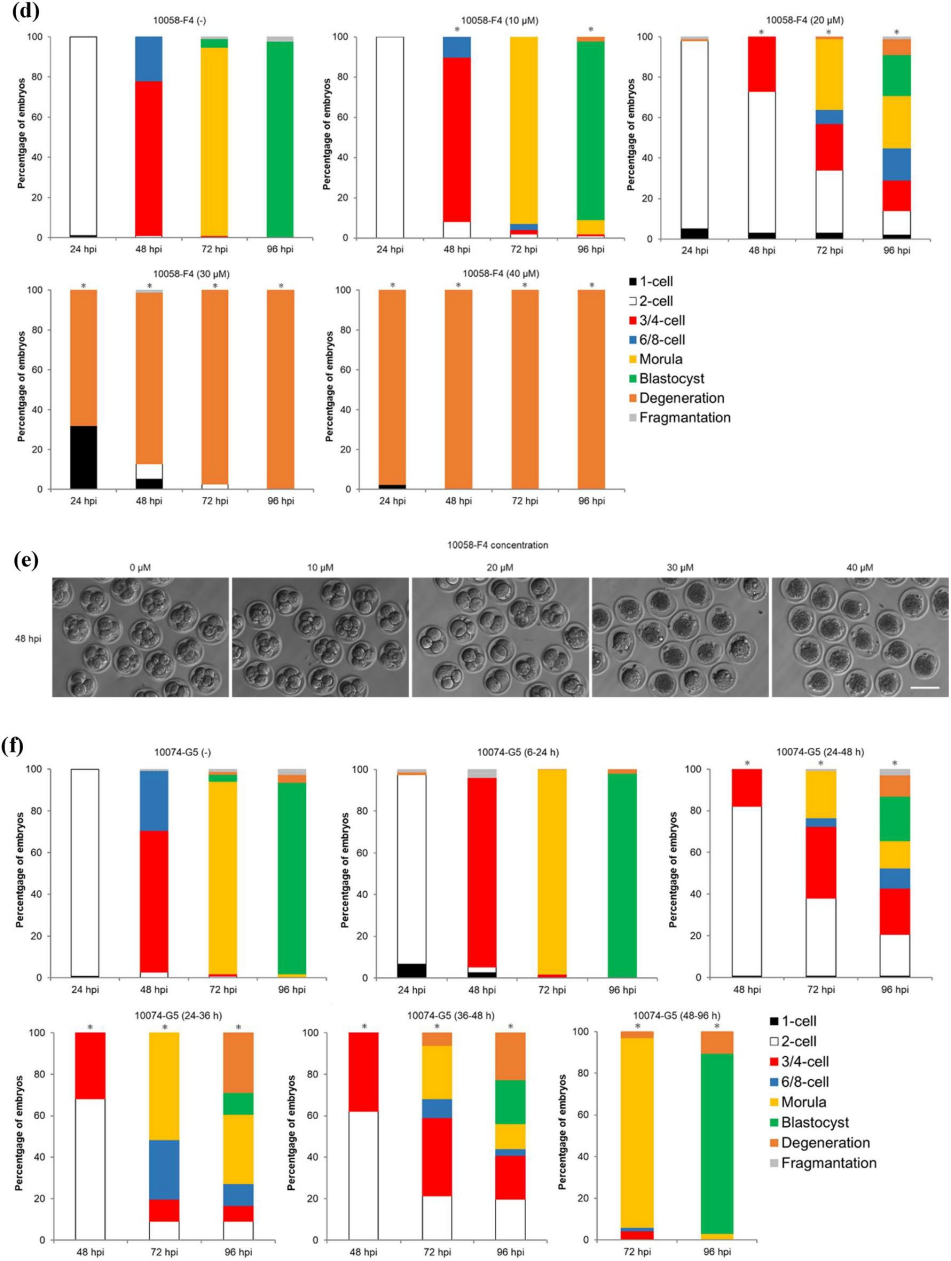

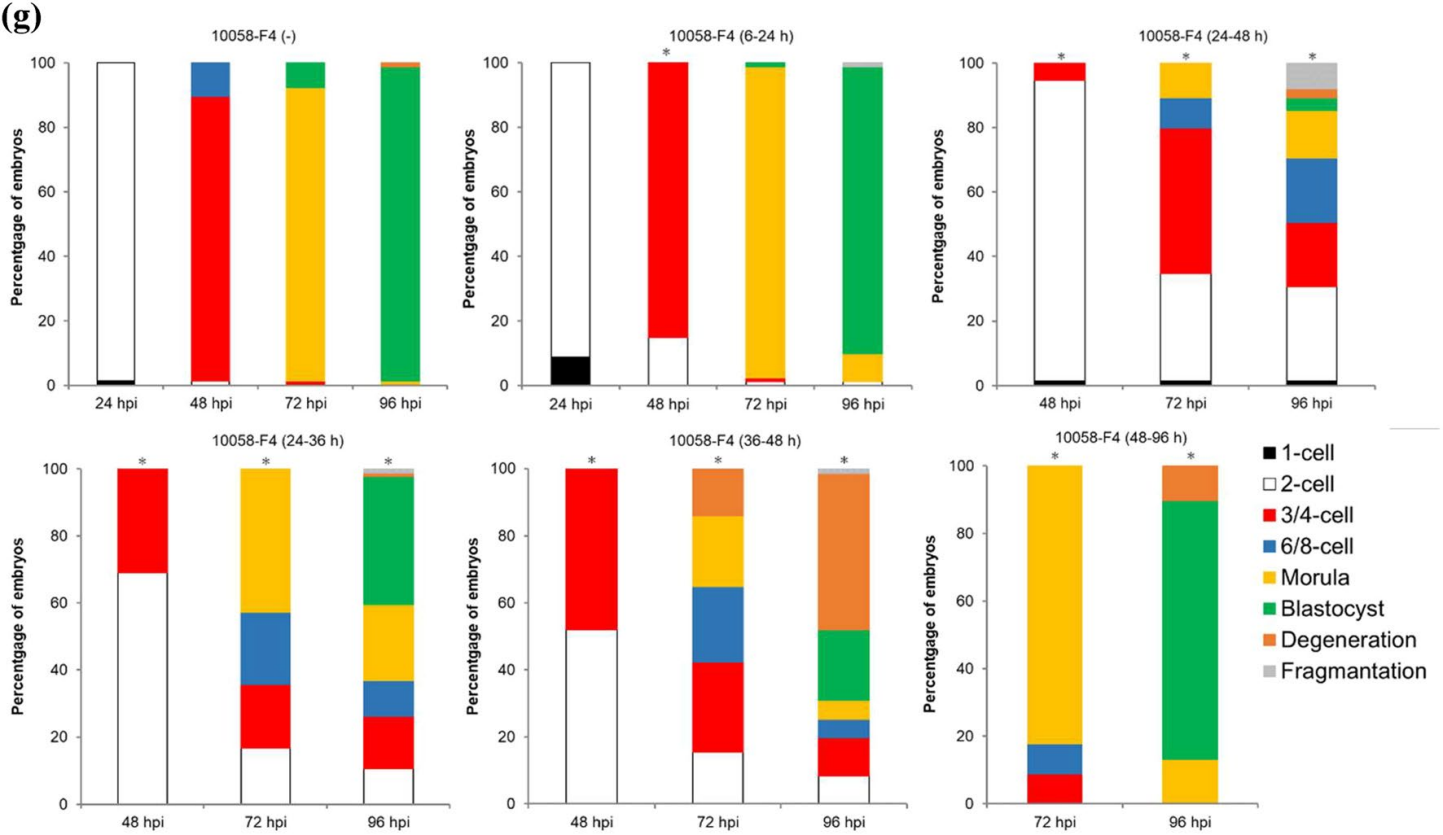
Developmental competence of embryos treated with inhibitors of MYC–MAX heterodimerization. (**a**) Percentage of embryos by developmental stage in 10074-G5 (−) embryos (*n* = 120) or embryos treated with 10074-G5 at the indicated concentrations (2 µM, *n* = 119; 4 µM, *n* = 119; 6 µM, *n* = 119; 10 µM, *n* = 20) from 6 to 96 hpi. 10074-G5 (−) indicates treatment with DMSO, the solvent for 10074-G5, between 6–96 hpi. (**b**) Representative images of 10074-G5 (−) embryos and embryos treated with 10 µM 10074-G5 from 6 hpi. Embryos were photographed at 24 hpi. Scale bar, 100 μm. (**c**) Representative images of 10074-G5 (−) embryos or embryos treated with 2, 4, and 6 µM 10074-G5 from 6 hpi. Embryos were photographed at 48 hpi. Scale bar, 100 μm. (**d**) Percentage of embryos by developmental stage in 10058-F4 (−) embryos (*n* = 96) or embryos treated with 10058-F4 at the indicated concentrations (10 µM, *n* = 99; 20 µM, *n* = 100, 30 µM, *n* = 78; 40 µM, *n* = 80) from 6 to 96 hpi. 10058-F4 (−) indicates treatment with DMSO, the solvent for 10058-F4, between 6 and 96 hpi. (**e**) Representative images of 10058-F4 (−) embryos or embryos treated with 10, 20, 30, and 40 µM 10058-F4 from 6 hpi. Embryos were photographed at 48 hpi. Scale bar, 100 μm. (**f**) Percentage of embryos by developmental stage in 10074-G5 (−) embryos (*n* = 157) or embryos treated with 6 µM 10074-G5 at the indicated periods (6–24 hpi, *n* = 56; 24–48 hpi, *n* = 145; 24–36 hpi, *n* = 66; 36–48 hpi, *n* = 66; 48–96 hpi, *n* = 67). (**g**) Percentage of embryos by developmental stage in 10058-F4 (−) embryos (*n* = 77) or embryos treated with 6 µM 10058-F4 at the indicated periods (6–24 hpi, *n* = 81; 24–48 hpi, *n* = 75; 24–36 hpi, *n* = 84; 36–48 hpi, *n* = 71; 48–96 hpi, *n* = 68). Statistical analysis was performed using the chi-squared test with Holm’s adjustment. The number of normal embryos at 24 hpi (≥ 2-cell), 48 hpi (≥ 4-cell), 72 hpi (≥ morula), and 96 hpi (blastocyst) were compared with DMSO-treated embryos. *P < 0.05.

To investigate the function of MYC protein in mouse preimplantation development, two structurally distinct small molecule inhibitors, 10074-G5 and 10058-F4, were used in this study that specifically bind to different regions within the helix-loop-helix domain of MYC and prevent MYC–MAX heterodimerization. Recently, a study using MYC inhibitors has been reported, but their study did not determine the optimal inhibitor concentration^14^. However, in this study, we first determined the optimal inhibitor concentration to be the lowest one at which development stops at the two-cell stage during 6–96 hpi of inhibitor treatment. To determine the optimal concentration of 10074-G5 and 10058-F4 with the fewest side effects, embryos were treated with various concentrations of these inhibitors (0, 2, 4, 6, and 10 µM for 10074-G5; 0, 10, 20, 30, and 40 µM for 10058-F4) at 6–96 hpi. Embryos treated with 10 µM 10074-G5 or 30 or 40 µM 10058-F4 degenerated before cleavage due to cytotoxicity (Fig. 2a–e). Because the rate of development to the four-cell stage of embryos treated with 6 µM 10074-G5 or 20 µM 10058-F4 was as markedly reduced as those treated with *Myc*-ASOs (96.2% for 10074-G5 [−], 16.8% for 10074-G5 [6 µM], 99.0% for 10058-F4 [−], and 27.0% for 10058-F4 [20 µM] at 48 hpi) (Fig. 2a,c,d,e), these concentrations were used as the minimum effective concentrations in subsequent experiments. To determine the exact time when MYC is required in the development of preimplantation embryos, embryos were treated with the inhibitors at 6–24, 24–48, 24–36, 36–48, or 48–96 hpi. Although inhibitor treatment at 6–24 and 48–96 hpi did not affect embryonic development, treatment at 24–36, 36–48, and 24–48 hpi reduced the rate of development to the four-cell stage, and delayed development was observed in some embryos after transfer to the inhibitor-free medium (Fig. 2f and g).

### Inhibition of MYC–MAX heterodimerization suppresses ZGA

**Figure 3:**
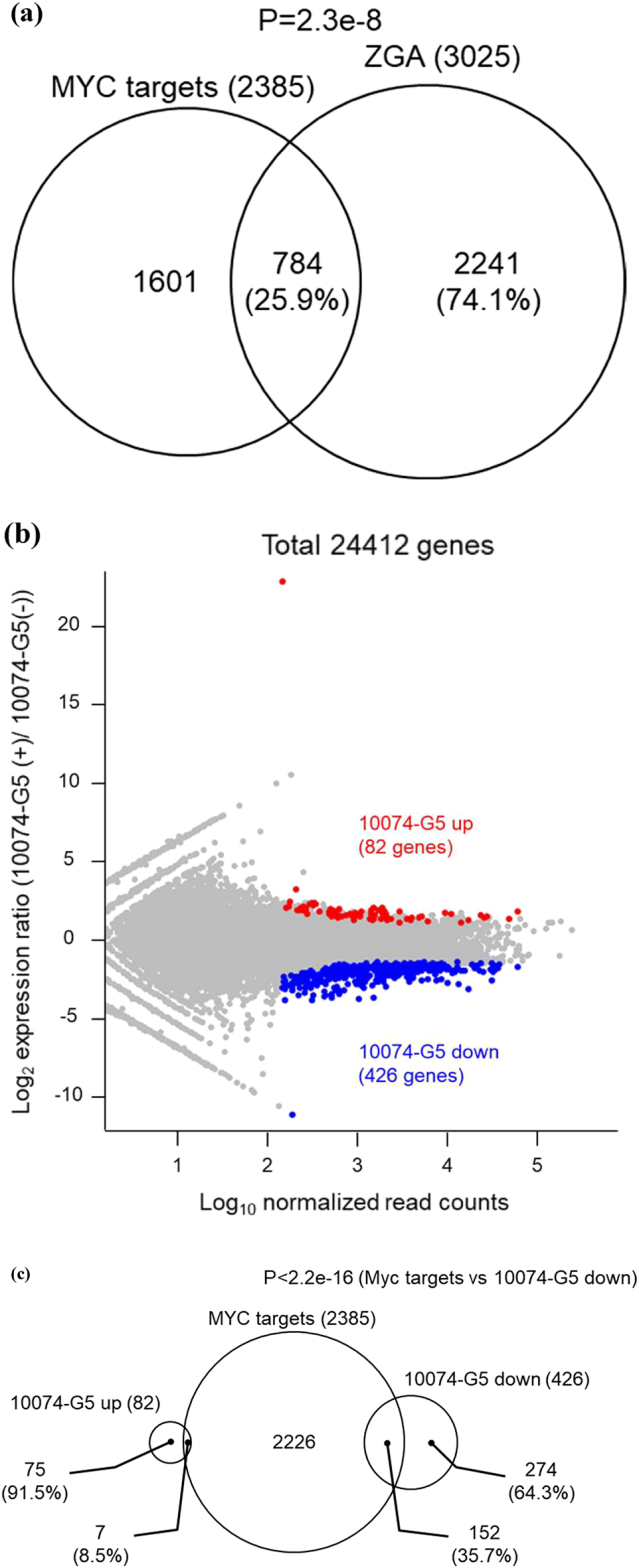

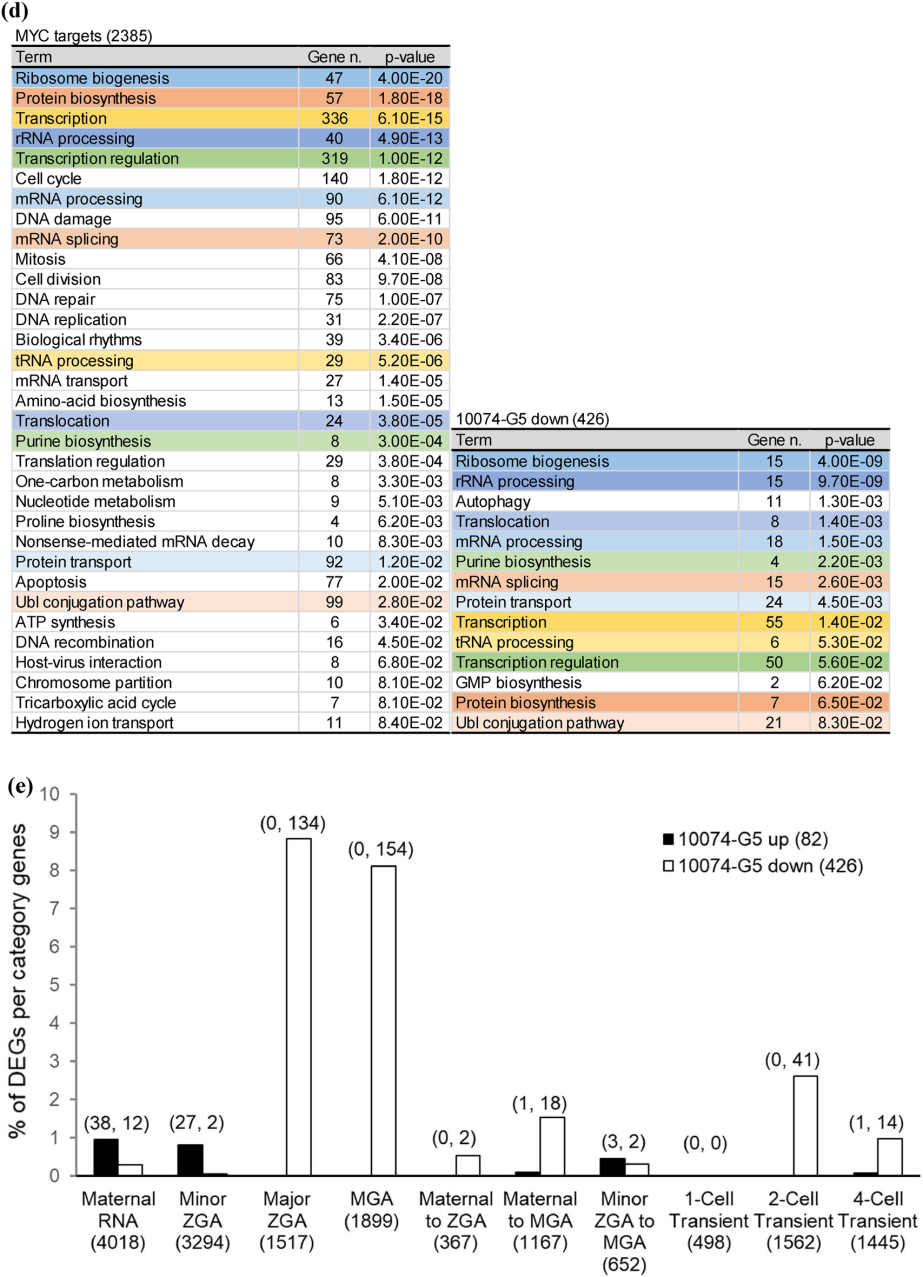

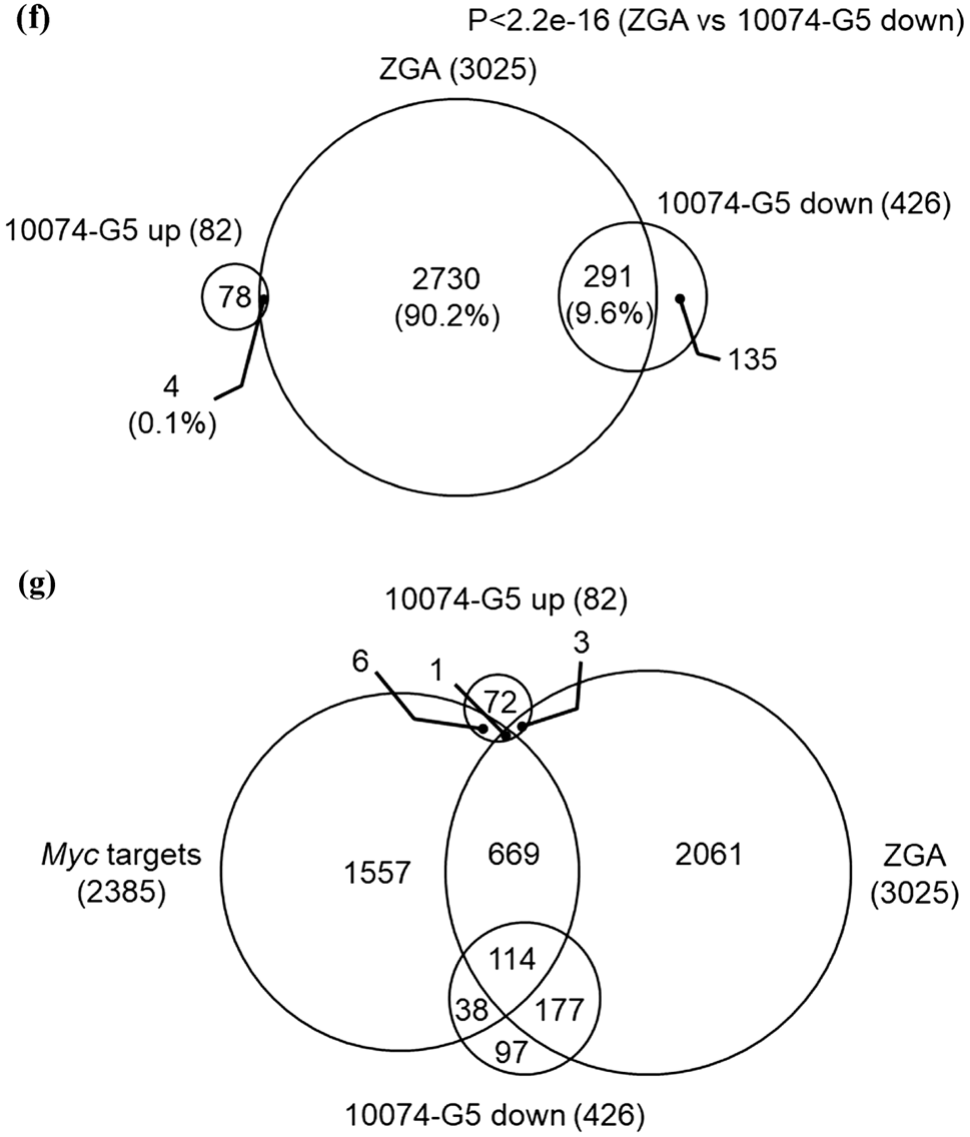
RNA-seq analysis indicates transcriptional aberration by inhibition of MYC–MAX heterodimerization between minor and major ZGA. (**a**) Venn diagram for MYC target genes and genes up-regulated during ZGA. *P-*values were calculated by Fisher’s exact test. (**b**) MA plot showing the gene expression ratios of 10074-G5-treated embryos to 10074-G5 (−) embryos and the average gene expression of all embryos. Among 24,411 genes, 82 were significantly up-regulated (red circles) and 426 were down-regulated (blue circles) in 10074-G5-treated embryos. Differentially expressed genes were defined as those with |log2FC|> 1 and adjusted *P*-value < 0.05 using DESeq2. (**c**) Venn diagram of MYC target genes and genes up- or down-regulated in 10074-G5-treated embryos. *P*-values were calculated by Fisher’s exact test. (**d**) Significant Gene Ontology terms for biological processes enriched with the MYC target genes and genes down-regulated in 10074-G5-treated embryos. Gene *n*. represents the number of related genes. (**e**) Percentage of genes up- or down-regulated in 10074-G5-treated embryos per genes of DBTMEE v2 transcriptome categories. The number of genes is indicated above the bars (up-regulated genes, down-regulated genes). (**f**) Venn diagram of genes up-regulated in ZGA and genes up- or down-regulated in 10074-G5-treated embryos. *P*-values were calculated by Fisher’s exact test. (**g**) Venn diagram of MYC target genes and genes up-regulated in ZGA and genes up- or down-regulated in 10074-G5-treated embryos.

Gene network analysis predicts that *Myc* is the gene controlling major ZGA^10,16,17^. We analyzed ChIP-seq data obtained from mouse embryonic stem cells (ESCs)^18^ and identified 2385 genes that were direct targets of MYC. A comparison of these genes with the 3025 genes that are elevated during ZGA (ZGA genes)^19^ revealed that 25.9% of ZGA genes are MYC target genes (Fig. 3a). RNA-seq analysis of late two-cell stage embryos treated with 10074-G5 at 6–36 hpi indicated that 82 genes were significantly up-regulated, and 426 genes were significantly down-regulated compared with control embryos (Fig. 3b). As *Myc*-inhibited embryos for RNA-seq, 10074-G5 treated embryos were selected because the most control embryos in the inhibitor experiment developed normally to blastocysts compared with the control embryos in the ASO experiment (91.7% for 10074-G5 [−] and 31.0% for NT-ASO at 96 hpi). Of the down-regulated genes, 152 (35.7%) were direct targets of MYC, and the biological processes shown in Gene Ontology analysis of MYC target genes were common with those of the down-regulated genes in 10074-G5-treated embryos (Fig. 3c and d). In addition, the transcription profiles of 10074-G5-treated embryos were compared with those of wild-type embryos listed in a public database (DBTMEE)^20^ (Fig. 3e). The results showed that many of the up-regulated genes in 10074-G5-treated embryos were maternal transcripts that were not degraded and genes that were expressed at the minor ZGA phase. In addition, 291 (9.6%) of the ZGA genes were suppressed by 10074-G5 treatment (Fig. 3f). Of the genes that were direct targets of MYC and up-regulated during ZGA, 114 were down-regulated by 10074-G5 treatment (Fig. 3g).

## Discussion

This study demonstrates that the transcription factor MYC is essential for early mouse embryogenesis by using *Myc*-targeting ASOs and MYC-specific inhibitors. We found that developmental arrest occurs at the two-cell stage when embryos are treated with ASOs or inhibitors from the one-cell stage. The functions of *Mycn* and *Mycl* are redundant to those of *Myc*, and embryos in which *Myc* is replaced with *Mycn* develop normally^21^. Although the inhibitors used in this study have been shown to be effective against MYCN and MYCL^22,23^, developmental arrest occurred at the two-cell stage even with ASOs targeting only *Myc*. These results suggest that only *Myc* is essential for early development. This idea is also valid because RNA-seq data analysis indicates that *Mycn* and *Mycl* expression is low in early development. Although Paria et al. showed that *Myc*-targeting ASO treatment from the two-cell stage results in embryonic arrest at the eight-cell/morula stage^12^, we found that some embryos were similarly arrested at the eight-cell/morula stage (10074-G5: 80.3% and 10058-F4: 33.3%) when two-cell embryos were treated with MYC inhibitors for a limited time period (24–36 hpi) in this study. This suggests that major ZGA at the two-cell stage induced by MYC was partially inhibited in embryos arrested at the eight-cell/morula stage by ASO treatment in the previous study and by the time-limited inhibitor treatment in the present study.

More recently, it was reported that treatment of mouse one-cell embryos with 10058-F4, the MYC inhibitor used in the present study, results in developmental arrest at the one-cell stage^14^. Abe et al. reported that inhibition of minor ZGA alone does not impair development from the one-cell stage to the two-cell stage but does impair development after the two-cell stage^24^. Therefore, we hypothesized that the inhibition of transcription factors such as *Myc* would not result in one-cell stage arrest. In our study, the optimal inhibitor concentration was defined as the concentration at which development stopped at the two-cell stage, similar to the results with ASOs, but when treated with a higher concentration of 10058-F4, as was the case in the study of Asami et al., developmental arrest or degeneration also occurred at the one-cell stage, suggesting that the effect of higher inhibitor concentrations on development may be an artifact. In our research, embryos developed normally into blastocysts when they were treated with the inhibitors at the one- to two-cell stage (6–24 hpi) or after the four-cell stage (48–96 hpi), also suggesting that the inhibitors specifically inhibit MYC without cytotoxic effects. Asami et al. performed RNA-seq using a library independent of the poly-A tail, whereas polyadenylated RNA-seq libraries were used in this experiment. It is probable that the difference of the libraries used affected the number of differentially expressed genes.

RNA-seq analysis of embryos treated with 10074-G5 at 6–36 hpi (from just after fertilization to the late two-cell stage) showed that only two minor ZGA genes were down-regulated, while 288 major ZGA and mid-preimplantation gene activation (MGA) genes were down-regulated. This indicates that MYC is required for the induction of major ZGA, as pointed out in previous studies involving gene network analysis^10,16^, and that the transcriptional aberration caused by MYC inhibition occurs between minor ZGA and major ZGA. This result is supported by a report showing that MYC proteins co-localize with nuclear speckles from the two-cell stage^11^, suggesting that MYC localizes to transcriptionally active regions around nuclear speckles and promotes transcription of the embryonic genome. In addition, activated β-catenin, a transcription factor of the Wnt pathway, localizes to the nucleus in one- to two-cell stage embryos. Tankyrase promotes the nuclear localization of β-catenin, but inhibition of tankyrase arrests mouse preimplantation development at the two-cell stage. It has been postulated that this is due to the inhibition of the nuclear localization of β-catenin, which inhibits the function of MYC, a direct target of β-catenin, thereby preventing ZGA^17^, and our results support this hypothesis.

Meanwhile, RNA-seq analysis indicated an increase in the number of genes classified as maternal RNAs, but this result was attributed to the lack of degradation of maternal transcripts. There were only 38 such genes, indicating that the effect of MYC inhibition on the degradation of maternal transcripts was relatively small. Gene Ontology analysis of MYC target genes and genes whose expression was down-regulated by MYC inhibition showed that both contain many genes involved in pathways such as ribosome biosynthesis, protein biosynthesis, and transcription. These results suggest that the downstream genes of MYC dominate the central pathways in major ZGA, and developmental arrest occurs because these pathways are disrupted.

Of the 2,385 direct MYC target genes obtained from ChIP-seq data in ESCs, only 152 (6.37%) were down-regulated by MYC inhibition at the two-cell stage. The genes whose expression was not decreased may be due to differences in MYC target genes between ESCs and early embryos. Indeed, among the top 500 genes that were highly expressed in dimethyl sulfoxide (DMSO)-treated control embryos, 167 (33.4%) were MYC target genes, and 44 (26.3%), a higher proportion than overall MYC target genes in ESCs, were down-regulated by MYC inhibition. In addition, based on the observation that when the relative amount of MYC is low, MYC binds only to high-affinity targets, and when the relative amount is high, MYC also binds to low-affinity targets^25,26^, it is possible that the difference in the relative amount of MYC between ESCs and early embryos was the reason for the low number of genes whose expression was decreased by MYC inhibition at the two-cell stage.

*Myc* is one of the proto-oncogenes commonly expressed in various types of cancer cells, and the feature that both cancer cells and early embryos lack cell cycle checkpoints and proliferate rapidly may be derived from the effects of *Myc*^27^. *Myc* is also one of the genes selected for the establishment of induced pluripotent stem cells because it maintains the pluripotency of ESCs, and the expression of four genes, including *Myc*, can reprogram fibroblasts into pluripotent stem cells^28^. However, it has been shown that two-cell-like cells are induced from ESCs when *Myc* is knocked out, suggesting that *Myc* prevents the transition from the pluripotent state to the totipotent state in ESCs^29^.

This study demonstrates that *Myc* is required at the two-cell stage in embryos, the time when the totipotent state is lost and the transition to the pluripotent state is initiated, suggesting that *Myc* also affects the differentiation potential of early embryos. Our understanding of the “essential” role of *Myc* in early embryogenesis will accelerate our understanding of cancer cells and induced pluripotent stem cells.

## Materials and methods

### Collection of oocytes, in vitro fertilization, and embryo culture

Eight- to 12-week-old ICR mice (Japan SLC, Shizuoka, Japan) were superovulated by injection with 7.5 IU equine chorionic gonadotropin (eCG: ASUKA Animal Health, Tokyo, Japan) followed 46–48 h later by 7.5 IU human chorionic gonadotropin (hCG: ASUKA Animal Health). Mice were euthanized by cervical dislocation by trained personnel following the American Veterinary Medical Association (AVMA) Guidelines for the Euthanasia of Animals (2020)^30^. No anesthetic agent was used. Cumulus-oocyte complexes were collected from the ampullae of excised oviducts 14 h after hCG injection and placed in 100 μL human tubal fluid (HTF) medium supplemented with 4 mg/mL bovine serum albumin (BSA: Sigma-Aldrich, St. Louis, MO)^31^. Twelve- to 18-week-old ICR male mice (Japan SLC) were euthanized by cervical dislocation, and spermatozoa were collected from the cauda epididymis and cultured for at least 1 h in 100 μL HTF medium. After preincubation, the sperm suspension was added to the fertilization droplets at a final concentration of 1.0 × 10^6^ cells/mL. At 6 hpi, morphologically normal fertilized embryos with two identified pronuclei were collected and cultured in potassium simplex optimized medium (KSOM) supplemented with amino acids^32^ and 1 mg/mL BSA under paraffin oil (Nacalai Tesque, Kyoto, Japan). All incubations were performed at 37 °C under 5% CO_2_. Embryos were incubated until 96 hpi.

### RNA extraction and RT-qPCR

Total RNA extraction and cDNA synthesis from oocytes were performed using a SuperPrep™ II Cell Lysis & RT Kit for qPCR (TOYOBO, Osaka, Japan). Synthesized cDNA was mixed with specific primers and KOD SYBR qPCR Mix (TOYOBO), followed by RT-qPCR amplification. The protocols for RT-qPCR and the establishment of transcript levels were performed as previously described^33^, and *Gapdh* or *H2afz* was used as an internal control. Relative gene expression was calculated using the 2^−ΔΔCt^ method^34^. Primer sequences used for RT-qPCR were as follows: *Gapdh*, 5′-GTGTTCCTACCCCCAATGTG-3′ (forward) and 5′-TGTCATCATACTTGGCAGGTTTC-3′ (reverse); *H2afz*, 5′-TCCAGTGGACTGTATCTCTGTGA-3′ (forward) and 5′-GACTCGAATGCAGAAATTTGG-3′ (reverse); *Myc*, 5′-CGTTGGAAACCCCGCAGA-3′ (forward) and 5′-TCCAGATATCCTCACTGGGCG-3′ (reverse).

### Immunocytochemistry and fluorescence analysis

To detect the localization of MYC, embryos were fixed with 4% paraformaldehyde in phosphate-buffered saline (PBS) for 20 min at 28 °C. The embryos were then treated with 0.5% Triton X-100 (Sigma-Aldrich) in PBS for 40 min at 28 °C. The embryos were blocked in PBS containing 1.5% BSA, 0.2% sodium azide, and 0.02% Tween 20 (antibody dilution buffer) for 1 h at 28 °C, followed by overnight incubation at 4 °C with a rabbit anti-MYC antibody (1:20,000; ab32072; Abcam Ltd., Cambridge, UK) in antibody dilution buffer. Subsequently, the samples were washed in antibody dilution buffer and incubated with an Alexa Fluor 594-conjugated goat anti-rabbit IgG secondary antibody (1:500; Thermo Fisher Scientific, Waltham, MA) for 1 h at 28 °C. After washing in antibody dilution buffer, the embryos were stained with antibody dilution buffer containing 10 μg/mL Hoechst 33342 (Sigma-Aldrich) for 20 min at 28 °C. Stained embryos were mounted on glass slides, and signals were observed using a fluorescence microscope (IX73; Olympus, Tokyo, Japan).

### Microinjection of ASOs

The following ASOs were purchased from Qiagen (Hilden, Germany): *Myc*-ASO-1, LG00828024-DDA; *Myc*-ASO-2, LG00828026-DDA; and NT-ASO, LG00000002-DDA. Approximately 3–5 pL of 10 µM ASOs was microinjected into the cytoplasm of zygotes at 3 hpi in HEPES-buffered KSOM. Microinjection was performed under an inverted microscope (IX73) equipped with a piezo injector (PMAS-CT150; PRIME TECH, Tokyo, Japan) and a micromanipulator (IM-11-2; Narishige, Tokyo, Japan).

### Inhibitor treatment

10074-G5 (Cayman Chemical Company, Ann Arbor, MI) and 10058-F4 (Cayman Chemical Company) are MYC inhibitors that bind to and distort the basic helix-loop-helix-ZIP domain of MYC, thereby inhibiting MYC–MAX heterodimer formation and inhibiting its transcriptional activity^35^. These two inhibitors are structurally unrelated and their cognate binding sites on MYC are distinct from each other. Embryos were cultured in KSOM containing 2, 4, 6, or 10 µM 10074-G5 or 10, 20, 30, or 40 µM 10058-F4 at 6–24, 6–96, 24–36, 24–48, 36–48, or 48–96 hpi. Control embryos were cultured in inhibitor-free medium that contained the appropriate amount of DMSO, the solvent used to prepare the stock solution of inhibitors.

### Publicly available data

The MYC ChIP-seq data were downloaded from ref.^18^ with the Gene Expression Omnibus accession number GSM1171648. Raw reads were aligned to the mm10 genome, using Bowtie 2 (ver. 2.4.5)^36^. Removing mapping duplicates and handling of sam and bam files were performed with SAMtools (ver. 1.10)^37^. The peaks were called using MACS2 (ver. 2.2.7.1)^38^.

### RNA-seq and data processing

Two-cell stage embryos (*n* = 10) in the control and inhibitor-treated groups were collected for RNA-seq library construction at 36 hpi. Two biological replicates were obtained from each group. Polyadenylated RNA-seq libraries were prepared by using a SMART-Seq v4 PLUS Kit (Clontech, Mountain View, CA). Indexed RNA-seq libraries were sequenced using an Illumina HiSeqX sequencer (paired end, 150 bp). The resulting sequence reads were mapped to the mm10 mouse genome, using STAR aligner software (ver. 2.7.10.a), and only uniquely mapped reads were used for subsequent processing^39^. Gene expression levels were calculated using RSEM (ver. 1.3.3)^40^ and differentially expressed genes were analyzed with the DESeq2 package (ver. 1.38.3)^41^. Differentially expressed genes were defined as those with |log2FC|> 1 and adjusted *P*-value < 0.05. Gene Ontology analysis was performed using the DAVID tool^42,43^.

### Statistical analysis

Developmental rates were analyzed using the chi-squared test with Holm’s adjustment. For gene expression analysis, RT-qPCR data were analyzed using one-way analysis of variance (ANOVA) followed by the Tukey–Kramer test. *P-*values < 0.05 were considered statistically significant.

### Ethical approval for the use of animals

All experimental procedures were approved by the Animal Research Committee of Kyoto University (permit no. R3-17) and performed in accordance with the committee’s guidelines. The study was carried out in compliance with the ARRIVE guidelines.

## Data Availability

The original data presented in this study are publicly available at: https://www.ncbi.nlm.nih.gov/geo/query/acc.cgi?acc=GSE233897.
